# The Management of Continued Fever

**Published:** 1852-03

**Authors:** 


### BUFFALO MEDICAL JOURNAL

AND

### MONTHLY REVIEW.

**VOL. 7.** MARCH, 1852. **NO. 10.**

### ORIGINAL COMMUNICATIONS.

### ART. I.—MANAGEMENT OF CONTINUED FEVER.

(Concluded.)

BY THE EDITOR.

The special indications pertaining to the *skin*, relate, first, to *increased heat, and dryness.* These symptoms are usually present, more or less, in Continued Fever. They denote a condition of the skin unfavorable for the exercise of its functions, and it is reasonable to suppose that thus, indirectly, they may prejudice the welfare of the patient, aside from compromising his comfort. The direct effect of an increased disengagement of caloric, moreover, it is not improbable, may contribute to some of the evils of the febrile state.

The most effective, and, in fact, the only refrigerating measures that possess much potency, are external applications, and these are *cold water* and *cool air.* Ablutions with cold water are usually very grateful to the sensations of patients affected with fever, and abate, frequently in a striking manner, the increased heat and dryness. The simplicity of the remedy causes it to be too lightly esteemed by attendants, and sometimes, perhaps, by physicians. It is really an important part of the treatment of a large proportion of fever cases. The face, body, and extremities, may be sponged, in succession, several times during the day, or as often as the heat and dryness returns. A faithful, judicious nurse may occupy a considerable portion of the time with these ablutions, to the advantage of the patient. Should *cold* water occasion uncomfortable sensations (which must rarely, if ever, be the case) tepid, or even warm water will secure, by evaporation, part of the refrigerating effect. The evaporation will be more rapid if spirit be added to the water. Cologne, or other perfumed spirits may be employed for this purpose, as a matter of taste, or elegance. The ablutions (with water) should extend to the mouth and teeth. The appearance, comfort, and, in some degree, the welfare of the patient are promoted by removing the deposits on the teeth and lips (sordes), and cleansing the tongue and mouth, so far as practicable, from the vitiated secretions.

Cold water taken into the stomach exerts a refrigerating effect on the skin, and the system at large. Patients should be allowed to drink freely, the only restraint required being not to take at a single draught sufficient to distend the stomach and occasion distress or vomiting. The refrigerating effect of cool air is important. This is one of the useful ends of free ventilation. To secure this end the patient should be lightly covered, and ventilation beneath the bed clothes attended to.

The skin being endowed with important excretory functions, the supposition that a morbid material may be eliminated through this channel, provided the humoral pathology of fever be adopted, is certainly not irrational. The notion that fevers sometimes arrive at a favorable termination by means of a critical cutaneous excretion, has for a very long period been entertained, and is still in vogue with the profession, and especially with the public. Upon this idea is based the indication for measures designed to induce diaphoresis in Continued Fever. Is this a valid indication? So far as the results developed by my analyses bear upon this question, they do not furnish evidence for the affirmative. These results show that increased perspiration, or sweating, although occasionally coincident with a favorable change, is oftener not immediately followed by convalescence, or a distinct amelioration of symptoms. This being the case, the chances are unfavorable to an expectation of benefit from diaphoresis, were it practicable to be induced at will, unless it be more beneficial when it results from remedial measures than when it occurs spontaneously, which is not a very probable supposition. The most rational inference from the facts just mentioned is, that when sweating directly precedes or concurs with convalescence, or marked improvement, it denotes either a coincidence of phenomena having no connection of cause and effect, or that the sweating is one of the results of the favorable changes in the progress of the disease due to other causes. In the latter view the occurrence of sweating is a sign or token, instead of a means, but as it occurs as often without any such connection, it has little or no significance in this aspect, unless, at the same time, other symptoms denote improvement, or herald convalescence. Reasoning in this way there seems little encouragement to endeavor to excite the excretory function of the skin in the management of Continued Fever. Experience, however, may lead to a different conclusion, but I have no facts to offer bearing on this point, except the few observations already presented on the use of the wet sheet, the effect of which is mainly to promote diaphoresis.

The *Genito Urinary System* cannot be said, with our present knowledge, to furnish special indications for treatment, excepting attention to the bladder to obviate over accumulation of urine. The latter point is not to be overlooked in the management of the disease. The blunted perceptions of the patient may prevent, not only due attention on his part to the evacuation of the bladder, but the consciousness of suffering from distension. Hence, it is important to ascertain daily the quantity of urine passed, and to explore the hypogastric region whenever there is reason to suspect retention. The kidneys, as well as the skin, from an important excretory outlet, which may possibly be concerned in the elimination of matter upon which the continuance of the disease more or less depends. The secretion of urine in Continued Fever is generally scanty, and some observers have noticed, shortly before, or at the time of convalescence, the quantity to become more abundant, and containing an increased proportion of lithic acid, or the lithates. It does not, however, appear to have occurred to practitioners to endeavor to effect, by the use of diuretics, ends similar to those which are intended to be fulfilled by diaphoresis. All that is to be said on this point must, of course, be wholly conjecture. A collection of facts showing the results of experience is desirable, and the study of the changes which the urine undergoes in Continued Fever would perhaps tend to encourage the trial of remedies designed to affect the quantity and composition of this secretion.

The indications which I shall distinguish as *general*, either relate to the management of the disease without exclusive reference to any particular class of symptoms, or are deduced from the ensemble of symptoms. The *general indications* embrace, *first, sanitary conditions.* The hygienic treatment of Continued Fever is of prime importance. If compelled to elect between it and the employment of medicinal measures, in the majority of cases it would probably be the better policy to relinquish the latter. Remedies, be they ever so efficient and judicious, cannot compensate for the absence of favorable sanitary conditions. This follows as an inference from considerations already presented. Inasmuch as we do not claim to exercise any direct control, by means of medical art, over the morbid processes constituting the febrile state, and, consequently, do not address our remedies to the disease *per se*, the reliance for recovery must be mainly upon the self-adjusting power of the organism — a truth applicable to other affections than Continued Fever. It could hardly be otherwise, than that circumstances most conducive to the welfare of the economy in a state of health, in other words sanitary conditions, are of the first importance with reference to restoration.

The more important points belonging to the hygienic management pertain to temperature, ventilation, cleanliness, and attention to the various wants of the patient. Sufficient space to receive an abundance of wholesome air for respiration, etc., is a sanitary condition second to none other. In private practice, whenever practicable, a spacious apartment is to be selected. Frequently, through ignorance on this subject, patients are found occupying bedrooms too contracted to permit a proper supply of pure atmosphere. In hospitals the proportion of the area of the wards to the number of inmates should not fall below the limits which health requires. At least five hundred cubic feet should be allowed for each patient. These points, which are, of course, of more or less importance in the management of all diseases, claim as much attention in cases of fever as of any other affection.

The sick apartment, or ward, is to be kept at a comfortable temperature, care being especially taken to guard against an excess of heat.

Free ventilation is highly important. Adequate provisions for this end, especially in hospitals, demand attention. But, in addition to these, the doors and windows should be thrown open, in suitable weather, several times daily, to secure a more complete renewal of the atmosphere.

Cleanliness requires ablutions of the body, and frequent changes of the bed clothes and body linen. Whenever circumstances permit, it is an excellent plan to have two beds provided, and to transfer the patient daily from the one to the other. The change is in this way more complete, and effected with the least inconvenience, fatigue, and risk.

Attention to the wants, even where they are not expressed, is requisite. The condition of the mind is such that it becomes necessary to take pains to ascertain necessities, and sometimes to judge of their existence without reference to information given by the patient. Drink, for example, is to be given at the discretion of the attendant without reference to any spontaneous expression of thirst, or, sometimes, without regard to the response to the inquiry whether it be desired or not. Efforts at appropriate times to evacuate the bladder and bowels, are to be suggested and encouraged, etc.

The co-operation of an intelligent, judicious nurse, is in no disease more indispensable to successful management than in Continued Fever. This is the more essential in proportion to the peculiar degree of importance belonging to the measures which, of necessity, must be left in some measure to their discretion as well as fidelity; and, in many cases of Continued Fever, upon measures of that class, viz., hygienic regulations, diet, etc., the success of treatment mainly depends. Not a few patients with this disease are lost through the incompetency, carelessness, or perverseness of those upon whom the physician is obliged to rely for carrying out the plan of management which he directs.

The *second* of the *general indications* is to *sustain the powers of the system through the febrile career.* This language is somewhat indefinite, owing to want of precision in our knowledge of the intimate nature of the forces by which the organism resists, and recovers from disease. Its practical significance is, however, sufficiently plain. In importance, sustaining measures rank next to those belonging to hygiene. The disease has its intrinsic limitations. It runs a circumscribed career; and the great object of management, in most cases, is to assist in bringing it course to a favorable termination The self-adjusting, restorative energies of the system, accomplish the work. To economize, sustain, and develop these energies, placing the patient under circumstances most favorable for their operation, is the chief duty of the physician.

Continued Fever may end fatally, not from the occurrence of lesions incompatible with recovery, but simply because the powers of the system fail before the disease reaches the termination of its career. If the patient can be sustained for a sufficient period, he is safe. It is not uncommon for active agencies to be required to obviate an immediate and strong *tendency to death* — the issue appears to hang upon a thread — yet, the sustaining measures prove efficient, the crisis is passed, and in a short time the patient passes from a state involving the greatest possible danger into convalescence. These facts exemplify the importance of the general indication under consideration.

The sustaining measures comprise *stimulants*, *tonic remedies*, and *nutriment.* These, almost invariably, are indicated in combination. I will, however, offer a few remarks on each under a distinct head.

*Stimulants.* The pathological views which recognized local inflammation as the proximate cause of fever, or an almost constant element of the disease, led practitioners to employ stimulants with great caution. They were administered only when the danger from exhaustion was imminent. Apprehension of commencing their use too soon (which was deemed fraught with danger) occasioned frequent delay until the patient was about to succumb, when little hope remained of benefit from a change of treatment. Great timidity in prescribing this class of remedies even now exists, more or less, partly because it is not easy in medical practice to escape from the influences of tradition and of habit, and, in part, because the pathological views just referred to are still in vogue to a considerable extent.

My observations have been sufficient to convince me that, of the great majority of cases of Continued Fever, stimulants may with advantage enter into the management; and that they often form a highly important part of the treatment. They may be prescribed whenever the symptoms indicate the propriety of sustaining measures in general. Feebleness, compressibility, and great frequency of the pulse, diminished impulse and sounds of the heart, coolness of the external surface and copious perspiration, muscular debility, and low delirium — these are the more prominent of the symptoms which point to the importance of supporting the powers of the system. In proportion to the degree of weakness as denoted by these symptoms will be the urgency of the indication, and the extent to which sustaining measures are to be carried.

The stimulants which may be employed, in conjunction with tonics and nutriment, are those of the diffusible class, wine and spirits, and other excitants such as camphor and ammonia. The diffusible, or alcoholic class is most uniformly applicable, and the others may be superadded if it be desired to render the measures still more efficient. Spirits have appeared to me to be preferable to wine, and brandy has been the article I have generally employed.

The period in the progress of the disease when stimulants are called for, will, of course, vary considerably in different cases. They may in some instances be demanded almost from the commencement. Generally speaking, they will be useful after the first week. So, also, as respects the amount to be prescribed, there is no uniformity, and consequently no routine rules can be laid down. This, as well as the time when it is advisable to enter on the use of stimulants, is to be determined by the circumstances proper to individual cases. The quantity to which I have been accustomed to give in the average of cases, at a time, is about half an ounce, or a table spoonful. This dose may be repeated with intervals varying from half an hour to six hours, according to the evidences of the need of stimulants, and their effects. To produce a stimulant action upon the digestive organs the brandy should not be too much diluted. An equal quantity of water is sufficient.

With proper care and attention, I feel assured that fears lest the patient should receive injury by commencing too soon the use of stimulants, are groundless. When there is room for doubt as to their propriety, they may be given at first cautiously, allowing sufficient interval to watch their effect. The remedy can be suspended before any considerable harm is done should it be found to be inappropriate. There is much more risk involved in deferring the use of stimulants too long. Just in proportion to their usefulness is, of course, the injury to the patient the longer they are withheld. The time that is lost cannot be regained. It is therefore far better to commence too early than to incur any needless delay; and if the indication be not perfectly clear it is more judicious to test the question by a cautious trial than to wait for farther evidences of exhaustion. Judging from my own observations, if commenced when the indication is a matter of question, and given with due moderation, they will seldom be discontinued. It is easier to maintain strength, than to restore it; and this, as it seems to me should be the end of sustaining measures. Agreeably to this view it is preferable to anticipate the period when the powers of the system evince marked prostration, than to lose any time afterward.

Fear of employing stimulants in fever, is based on their usual effect in health. Their action, however, is quite different when they are indicated to sustain the vital powers. They do not then, in fact, act as stimulants. They do not, even when given largely, disturb the intellect, occasioning any of the phenomena of intoxication; and the influence on the circulation is such that their operation may be called sedative. I have in several instances, observed the pulse, when its frequency was greatly increased, numbering from 130 to 140, fall rapidly while the patient was taking half an ounce of brandy hourly, or half-hourly. An illustration of this strikingly sedative effect on the pulse has fallen under my notice within a few days of the time I am penning these remarks. A pulse much increased in frequency, in fact, furnishes a special indication for stimulants.

Aside from the circulation, which should improve under the beneficial operation of stimulants, the evidences of its good effects consist in better warmth and mellowness of the surface, lessened delirium, refreshing sleep, moisture of the tongue, etc. If a more favorable condition does not speedily follow, it is by no means to be inferred that the remedy is uncalled for, and should be discontinued, so long as there is no aggravation of symptoms denoting unpleasant consequences.

As respects other excitant remedies, my experience is mostly limited to the two articles mentioned, viz., *camphor* and *ammonia*. Camphor is supposed by some observers to be especially suited to cases characterized by ataxic symptoms, such as muttering delirium, insomnia, subsultus. The impression derived from some trial of it under these circumstances is, that its value has been overrated. It has not appeared to me to fulfill the expectations formed from the recommendations of others.

The *carbonate of ammonia* is specially suited to cases in which it is desired to produce an effect on the circulation. These and other remedies of an analogous character cannot be considered substitutes for diffusible stimulants, but they doubtless are more or less valuable as adjuvants.

If the view which I have submitted on the subject of stimulants differ from those entertained by many, if not most practitioners, it is not necessary to inform the reader that they are neither original, nor entitled to the character of singularity. In advocating the importance of this class of remedies in the treatment of Fever, I do but contribute my humble testimony to that of several distinguished writers, among whom the names of Drs. Graves and Stokes, of Dublin, are particularly to be mentioned.

*Tonics.* The indication to *sustain the powers of the system* would seem to call for remedies distinguished as tonics. That they are useful in the treatment of Continued Fever, is altogether probable. Their utility, however, is limited by the fact that their tonic influence is cumulative: an appreciable effect requires an aggregate of doses extended over a considerable period. They are not sufficiently prompt in their action to be of great service in fever. They are better suited to chronic maladies.

The tonic remedies best adapted are the bitter infusions and quinia, especially the latter, which I have frequently prescribed in cases of fever, but I am not prepared to say how much importance is to be attached to it. I cannot conceive of tonics doing harm, whenever sustaining measures are appropriate, except possibly by offending the stomach, and, thus, counteracting the end to which to be useful they must be subservient, viz., the ingestion and assimilation of nourishment.

*Nutriment.* The powers of the system cannot be effectually sustained without the assimilation of aliment. If we would fulfill the general indication under present consideration, then, nutriment must be received, digested, and appropriated by the economy. Are the assimilatory processes suspended n Continued Fever? Observations warrant an answer to this question in the negative. Nutriment, given in this disease, may be retained, without being followed by diarrhœa, the evacuations even being in some instances natural in appearance. Under these circumstances it must be digested and appropriated. I have witnessed a marked development of strength during the continuance of the febrile career. A patient who came under my charge after fever had existed for several days, and who had taken nothing but rice water, presented evidences of great prostration; but under an improved system of dietetics, although the duration of the disease was long, there was a decided gain in muscular strength, as well as in other particulars, notwithstanding the febrile career persisted. If the powers of the system may thus be developed during the continuance of fever, they can be preserved, or, in in other words, sustained by means of nutrition. Upon these facts the importance of nutriment in the management of Continued Fever is based. Relatively, this class of sustaining measures is the most important. It is mainly with reference to promoting the digestion and appropriation of nutriment that stimulants and tonics are useful. Except as subsidiary to assimilation, the good effects of the latter can only be transient, for it is alone through the addition of fresh alimentary supplies that the organism acquires renewed energy and strength. The idea of local inflammation as the proximate cause, or a constant element of Continued Fever, which has rendered practitioners distrustful of stimulants, has also prevented the importance of alimentation from being appreciated. With the attention fixed on inflammation, the *antiphlogistic regimen* was thought to be indispensable. But when it is considered that a great source of danger in fever cases is in the liability that the powers of life will give way, before the limited career of the disease is reached, and that a leading indication in the treatment is the one under consideration, viz., to sustain the system, it must be apparent that the only rational ground for doubt is whether the condition of fever is incompatible with the digestion and assimilation of food.

In the management of the disease, my practice is uniformly, after the lapse of a few days, at farthest the first week, to commence the administration of highly nutritious aliment. The forms of nourishment which I have thus far selected, are the *essence of beef*, and what is known commonly in this part of the country as *milk porridge*, consisting of milk scalded, with the addition of a little wheat flour. By the essence of beef is meant the pure juice of the meat, extracted by putting lean portions in a vessel (usually a stone or glass bottle) which is placed in boiling water, no water being added to the meat. The fluid thus obtained is quite different from what passes under the name of *beef tea*, which is merely warm water flavored with *osmazome*, having very slight nutritious properties. Occasionally I have given, in addition to the two articles just mentioned, strong chicken soup.

I direct these forms of diet to be administered systematically, without reference to the appetite or wishes of the patient, generally in alternation, small quantities being given at a time, at short intervals. The quantity of nutriment to be taken in a given time is, of course, to be regulated somewhat by the urgency of the indication for sustaining measures. It should correspond generally with the amount of stimulants, both being conjoined for the same end. The systematic administration of nutriment is also to be commenced at the period in the progress of the disease when stimulants are called for, both being simultaneously entered upon. Explicit specifications as to quantity, and intervals, are to be made in individual cases by the practitioner, especially in private practice, but routine rules cannot be laid down which will apply indiscriminately to all instances. In the average of cases I have been accustomed to direct from half an ounce, to an ounce, of the essence of beef, hourly, and the milk porridge in somewhat larger doses, making up the quantity, if, for any reasons, they are not given regularly at so short intervals. I state this as an approximation to a general rule of diet as respects quantity.

The articles of diet mentioned are selected in consequence of their containing nutricious principles in a concentrated form, and of a digestible character, being, at the same time, easily prepared, and administered.

They are sometimes taken by patients without reluctance, and occasionally are apparently acceptable to the palate. Oftener, however, they are undesired, and frequently considerable disinclination is manifested. The latter may proceed, in a measure, from the indisposition to make any effort, or to be disturbed, which belongs to the disease, rather than from a repugnance occasioned by the idea of food; but, in some instances, the antipathy is apparently not less than to doses of medicine.

As already stated, the course I have pursued is to have the nutriment administered, not only irrespective of any expressions of desire by the patient, but without reference to his appetite, and even when taken with disgust. If the sense of hunger, the appetite, and the taste are competent to govern the ingestion of food in a state of health, this is not true of Continued Fever. The proper exercise of these functions involves normal mental perceptions. In health, the mind feels certain impressions, derived from within the organism, by which the desire for food, or hunger, expresses the demand of the system for fresh supplies. The mind, in health, experiences pleasure from the ingestion of aliment, in other words from the indulgence of appetite, and is capable of discriminating appropriate kinds of food by the sense of taste. All this is changed in fever. The mental perceptions then fail to respond to the wants of the system; the mind lacks sufficient healthful activity for appetite or taste. The system, nevertheless, needs the addition of new materials for calorification and nutrition, and the power of assimilation, although impaired, is not wholly lost. The indication for nutriment, in short, exists, but the criteria pertaining to the consciousness of the patient by which this indication may be measured in health, are perverted or suspended by the disease, so that it becomes necessary to fulfill the indication without consulting the mental functions, viz., hunger, appetite or taste, which were doubtless designed to secure an adequate supply of proper aliment in health.

This, briefly, is the reasoning which leads to the propriety of administering nutriment in Continued Fever. But facts bearing on the subject, have more force than reasoning. The dietetic plan which has been described, I have pursued sufficiently to test, at least, its freedom from danger, or unpleasant consequences. I have not observed any apparent injury from it in a single instance, and I am persuaded that it not only tends to diminish the liability to a fatal termination by asthenia, but that the patient passes through the disease with less expenditure of vital strength, and recovers more rapidly and completely after convalescence is established.

The foregoing views relating to nutriment are by no means novel, although there is reason to think that they have been adopted by practitioners to a limited extent only. Dr. Graves, of Dublin, who dwells emphatically on this point in the management of fever, remarks, that if he has had more success than others in the treatment of the disease, it is owing, in a great degree, to the adoption of the advice of a country physician of great shrewdness, who advised him *never to let his patients die of starvation!** That patients may literally die of starvation when the disease is protracted, and the diet restricted to toast or rice water, does not seem to me improbable; and that the gravity of the disease — the prostration, delirium and other ataxic symptoms, etc., are often due, in no inconsiderable degree, to innutrition co-operating with the essential morbid condition in fever, I am fully convinced.

In conclusion, of the two types of Continued Fever, *Typhus* and *Typhoid*, in the former, as a general remark, measures designed to sustain the system are required more uniformly, oftener with urgency, and at an earlier period in the progress of the disease.

---

* System of Clinical Medicine, by Graves & Gerhard, Am. Ed.

*Treatment of Complications.* I shall notice only the more important of the complications which have occurred in the cases that have come under my own observation, viz., *Pneumonitis*, *Peritonitis*, *Parotiditis* and *Apoplectic Coma.*

*Pneumonitis.* It is obvious that in treating any inflammatory complication, the practitioner is not to have referred exclusively to the local affection. A consideration to be borne in mind is, that if the inflammatory affection be subdued, the patient has, nevertheless, to pass through the febrile career. It is also to be considered that there is less energy in the restorative processes, when fever is present, than if the local affection existed alone. Therapeutical measures which might be appropriate were fever not present, if they compromise the ability of the organism to withstand and surmount the febrile disease, may be highly improper. In all cases, remedies are to be employed under the restrictions imposed by due attention to the existence of Continued Fever, irrespective of the application. In the treatment of pneumonitis, as a primary affection, what is commonly known as the antiphlogistic plan of treatment is generally pursued, comprising general and topical blood-letting, antimony, mercurials, counter-irritation, and low diet. Now, in deciding whether the same method of treatment is admissible, or the extent to which it is to be pursued in the treatment of pneumonitis occurring as a complication of fever, we are to take into account the probable effect not alone on the pulmonary affection, but upon the condition of the patient with respect to the fever. Will it be likely to affect unfavorably the progress of the latter disease? To what extent may it be safely and judicially carried with reference, on the one hand, to the fever, and, on the other hand, to the inflammation? The practical bearing of these questions is sufficiently apparent. If, for example, blood-letting be the particular remedy proposed to be employed, its propriety, or the extent to which it is to be carried, will depend upon whether its usefulness in abating the severity of the pneumonitis be overbalanced by its diminishing the power of the system to sustain the duration of the febrile career. This conservative principle is applicable to the treatment of any, and all the complications of fever, as well as the particular affection under present consideration. According to it proper weight in all instances, will doubtless lead to quite different modes of practice, in individual cases, but, in general, the result will be, that the measures suited to primitive pneumonitis will by no means be uniformly indicated in the management of this affection occurring as a complication in Continued Fever, and, when admissible, it will be to a more or less limited extent.

It is hardly necessary to say, that various circumstances are to be taken into account in the treatment of pneumonitis superadded to fever, not less than when this affection is primary; such as the previous constitution of the patient; the extent to which the lungs are involved; the activity of the symptoms, etc.

Aside from these, there are certain features somewhat distinctive of the affection occurring as a complication of fever. The cases must be extremely rare in which the lungs become inflamed at the commencement, or during the early part of the febrile career. So far as my observations go, pneumonitis is not developed until the fever has advanced to the period when the antiphlogistic measures, that have been mentioned, are always to be employed with caution, lest the ability of the patient to bear up under the febrile disease be impaired. It is obvious that the later, in the progress of the disease, this complication occurs, the more is circumspection, in the employment of these therapeutical measures, called for. An activity of treatment, which might be judicious if the pneumonitis were developed at the onset of the fever, would be improper at a subsequent period, other things being equal.

Again, the inflammation of the lungs, developed during the progress of Continued Fever, seldom has that intensity or activity which belongs to acute pneumonitis as a primary affection. The rational symptoms are less prominent, and even the characteristic physical sign, the crepitant rale, is less constant and marked. The affection is more frequently latent. When devel- late in the disease, and especially in the Typhus type, (in which it oftener occurs,) it seems in a measure to come from the hypostatic congestion which is due to an enfeebled circulation and a morbid condition of the blood. The inflammatory action, under these circumstances, is of a very low grade. This has been sometimes distinguished as *pseudo-pneumonitis.*

In view of the foregoing facts and considerations, it would appear to be a rational conclusion, that pneumonitis complicating Continued Fever rarely calls for active treatment. General blood-letting is by no means advisable as the rule, but may perhaps be occasionally admissible. Topical bleeding is perhaps proper in a larger proportion of cases. Antimonials are to be employed with more circumspection than when the affection is primary. The propriety of counter-irritation by means of blisters is doubtful. Sinapisms, dry cupping, stimulating liniments, and the like, should constitute the revulsive measures. The appropriateness of mercury is questionable. The reader has doubtless observed that I have made no allusion to this remedy heretofore in connection with the treatment of fever. It was formerly very generally employed, and is still in vogue, to some extent, in the management of this disease. I believe, however, it may be said, that, by the most judicious practitioners at the present time, it is not considered necessary, or useful, in the treatment of fever uncomplicated by local inflammation. The antiplastic effect attributed to mercury would hardly seem indicated in a disease which modern researches have shown to involve, as a constant element, diminution in the fibrinous constituent of the blood. An increase of this constituent obtains in inflammations, and, hence, a rational indication for the supposed antiplastic operation of mercury. Cases of fever complicated with local inflammations have been found to present an increase, instead of a diminution of fibrin, and the inquiry therefore may arise, if, under these circumstances, mercurials are not rationally indicated. I am not prepared to answer this question. My observations furnish no facts bearing on the apparent results of the mecurial treatment of Continued Fever with, or without pneumonitis or any inflammatory complication. With respect to the general views submitted on the subject of the treatment of the complication under consideration, I should remark that, so far as my experience goes, it has appeared to be in confirmation of their correctness.

In conclusion, a practical point not to be overlooked is, that the occurrence of pneumonitis, or any secondary inflammation, does not of necessity conflict with the indication to sustain the powers of the system through the course of the fever. Stimulants, nutritious aliment, and tonics are not to be deferred, or laid aside in consequence of the development of an inflammatory complication, provided they are called for by the symptoms or condition of the patient, irrespective of the local affection. On the contrary, the co-existence of local inflammation may render a supporting plan of treatment more important, in order to increase the ability of the organism to carry on the processes of restoration.

*Peritonitis.* The remarks on the treatment of Pneumonitis will, in general, apply to Peritonitis, or any local inflammation complicating Continued Fever. To dwell upon this affection would therefore be to recapitulate much that has been already said. Peritonitis may result from intestinal perforation, or it may arise independently of that accident. It cannot, however, be of frequent occurence without a special tendency forming a characteristic of the disease at certain times or places.

Peritonitis from perforation, which is peculiar to the *Typhoid* type, almost of necessity occurs late in the febrile career, or during convalescence. The active measures of treatment generally deemed admissible in primary peritonitis are out of the question. The plan which is rationally indicated, and, which has the sanction of experience, being, if I mistake not, the only plan which has proved successful, consists in the exhibition of opiates in as large doses as can be borne without narcotism, repeated at proper intervals for several days, the bowels being allowed to remain unmoved. The object of this plan is to arrest the peristaltic movements, and favor the adhesion of the peritoneal surfaces surrounding the perforation. Recovery depends upon this object being effected. The chances of success are, however, exceedingly small. The merit of first pointing out and exemplifying the advantages of treating cases of perforation with large doses of opium, belongs to Dr. Graves, of Dublin.

Peritonitis, without perforation, is a less serious form of this complication, and claims the same measures proper to this affection, with all the restrictions applicable to the treatment of any local inflammation occurring in connection with Continued Fever. This general remark comprises all that need be said under this head.

*Parotiditis.* This complication, as the results of the preceding analyses have shown, involves a special tendency peculiar to the disease at certain times or places. The affection occurs at different stages of the disease, and, sometimes, as a sequel, during convalescence.

In none of the cases characterized by inflammation of the Parotid that I have observed, could it be regarded in the light of a critical event, and, so far from relieving the condition of the patient, he was exposed thereby to a new source of suffering and danger. The inflammation, in the cases that have fallen under my observation, was decidedly phlegmonous in its character, suppuration occurring after the lapse of several days. Active treatment, with a view to prevent the formation of pus, as a general remark, is not judicious, owing to the little prospect of effecting the object, and the risk of compromising the powers of the system. As the formation and discharge of pus will be likely to continue for some time, proving a source of exhaustion, the indication for sustaining measures is increased by the presence of this complication. A few leeches, in some instances, at first, might tend to diminish the intensity of the inflammation. I did not, however, resort to this measure in any of the cases that have fallen under my observation. Warm fomentations, and cataplasms to the inflamed part, opiates to relieve pain, opening the abscess so soon as fluctuation is discovered, and, afterward, a liberal administration of stimulants, tonics and nutriment, constituted the measures addressed to this complication.

*Apoplectic Coma.* Coma, developed more or less suddenly, is an event liable to occur during the career of Continued Fever. Could this complication be prevented, or relieved by appropriate treatment, the fatality of the disease would be diminished, inasmuch as in a certain proportion of cases a fatal termination is due to this event. In the *Second Clinical Report* I have called attention to symptoms referable to the respiration which precede and accompany apoplectic coma, by means of which the occurrence of this complication may be anticipated, showing, also, that the seat of the affection is at the medulla oblongata, life being destroyed by apnœa arising from suspension of the functions pertaining to that portion of the nervous system, which preside over the respiratory acts. The pathological condition upon which apoplectic coma occurring in fever depends, is a subject open for discussion, and farther investigation. It would be out of place to consider this subject in the present connection, but the treatment must have some reference to the pathological views which are entertained. I have submitted the opinion that the complication is due to effusion within the arachnoid cavity.* If this be the correct explanation, the probability of success from any therapeutical measures, after sufficient effusion has taken place to induce a state of coma, is slight. The suspension of the functions of the medulla is due to the mechanical pressure from the presence of fluid. To relieve the condition of the patient, the resorption of this effused fluid, or a portion of it, is to be effected. The chances are against the attainment of this end soon enough to rescue the patient from apnœa. The measures which suggest themselves, and to which I have thus far resorted, are a blister to the nucha, revulsives to the extremities, and cold applications to the head, other measures being continued or adopted according to the symptoms.

Although an exceedingly dangerous complication, apoplectic coma is not necessarily fatal. An instance affording a striking illustration of recovery under the most discouraging symptoms, is given in the *First Clinical Report:* case of McDonald, see section devoted to symptoms referable to the nervous system.

*Treatment of Convalescence.* I do not propose to consider the details of the management of Convalescence. Every physician is familiar with the precautions to be observed during the period which intervenes between the expiration of the febrile career, and the recovery of the usual health and strength. In this interval, the system is peculiarly susceptible to injury from over exertion of the powers of body or mind, and imprudences of any kind. The common idea that relapse of fever arises from error of management, is questionable. The singular contrast exhibited in the reports of the two preceding analyses with respect to this sequel, tends to disprove this idea. The

---

* See remarks on effusion within the arachnoid cavity, by the author, in the April No. of the Buffalo Medical Journal, 1850.

recurrence of a brief febrile career would seem to arise from some intrinsic tendency peculiar to the disease at certain seasons.

There is one point, pertaining to diet, respecting which the views commonly entertained appear to me to be faulty. I refer to the apprehensions frequently, if not generally felt by practitioners, in allowing a nutritious animal diet early in convalescence. These apprehensions, which I am persuaded are imaginary, have probably arisen, from the habit of associating Inflammation with Fever. The susceptibility to disorder, or consecutive diseases, is probably owing to the enfeebled condition of the organism after passing through the febrile career. It is an object, therefore, in the management of convalescence, to effect a return of the accustomed constitutional vigor as speedily as practicable. The means for this end are, aliment of the most digestible and nutritious kind, tonics, stimulants, and hygienic measures, of which one of the most useful is early gestation in the open air. I have pursued this course in a sufficient number of cases to feel satisfied from experience that it is not only unattended by danger, but is conducive to the well being of the patient. I have never witnessed any unpleasant results from the effort to re-establish the strength of the patient by resorting at once to an animal diet, etc.

A great advantage of the sustaining plan of treatment during the febrile career pertains to convalescence. The patient emerges from the disease with the powers of the constitution less enfeebled, the liability to *sequelæ* is diminished, and the recovery is more rapid and complete, other things being equal, in proportion to the pains taken to preserve, up to the date of convalescence, undue reduction of strength.

